# Making HIV testing work at the point of care in South Africa: a qualitative study of diagnostic practices

**DOI:** 10.1186/s12913-017-2353-6

**Published:** 2017-06-17

**Authors:** Nora Engel, Malika Davids, Nadine Blankvoort, Keertan Dheda, Nitika Pant Pai, Madhukar Pai

**Affiliations:** 1 Department of Health, Ethics & Society, Research School for Public Health and Primary Care, Maastricht University, Postbus 616, NL - 6200 MD Maastricht, The Netherlands; 2 Lung Infection and Division of pulmonology and UCT lung Institute Department of Medicine, University of Cape Town, Anzio Road, Cape Town, 7925 South Africa; 3 Division of Clinical Epidemiology, Department of Medicine, McGill University and McGill University Health Centre, V Building, Royal Victoria Hospital, 687 Pine Avenue West, Montreal, H3A1A1 Canada; 4 McGill International TB Centre, Department of Epidemiology & Biostatistics, McGill University, 1020 Pine Ave West, Montreal, QC H3A 1A2 Canada

**Keywords:** Diagnostic practices, Point of care, HIV, South Africa

## Abstract

**Background:**

Point of care testing promises to reduce delays in diagnosing and initiating treatment for infectious diseases such as Human Immuno-deficiency Virus (HIV). In South Africa, decentralized HIV testing with rapid tests offers important lessons for point of care testing programs. Yet, little is known about the strategies of providers and clients to make HIV testing successful in settings short of equipment, human resources and space. We aimed at examining these strategies.

**Methods:**

This paper is based on a larger qualitative study of diagnostic practices across major diseases and actors in homes, clinics, communities, hospitals and laboratories in South Africa. We conducted 101 semi-structured interviews and 7 focus group discussions with doctors, nurses, community health workers, patients, laboratory technicians, policymakers, hospital managers and manufacturers between September 2012 and June 2013 in Durban, Cape Town and Eastern Cape. The topics explored included diagnostic processes and challenges, understanding of diagnosis, and visions of ideal tests. For this paper, the data on HIV testing processes in clinics, communities and hospitals was used.

**Results:**

Strategies to make HIV testing work at point of care involve overcoming constraints in equipment, spaces, human resources and workload and actively managing diagnostic processes. We grouped these strategies into subthemes: maintaining relationships, adapting testing guidelines and practices to stock-outs, to physical space, and to different clients, turning the test into a tool to reach another aim and turning the testing process into a tool to enhance adherence. These adaptive strategies are locally negotiated solutions, often ad-hoc, depending on personal commitment, relationships, human resources, physical space and referral systems. In the process, testing is redefined and repurposed. Not all of these repurposing acts are successful in ensuring a timely diagnosis. Some lead to disruptions, unnecessary testing or delays with at times unclear implications for quality of diagnosis.

**Conclusion:**

Tests shape relationships, professional roles and practices of users at point of care. At the same time, testing processes are dynamic and test results and processes take on new meanings for clients and providers. These insights are crucial for understanding the contexts within which diagnostic devices and policies need to function.

**Electronic supplementary material:**

The online version of this article (doi:10.1186/s12913-017-2353-6) contains supplementary material, which is available to authorized users.

## Background

Global health experts agree that point-of-care (POC) testing in communities, home settings and primary healthcare centers has immense potential to reduce delays in diagnosing and initiating treatment for diseases like Human Immuno-deficiency Virus (HIV) [1, 2], tuberculosis (TB) [3, 4], syphilis [5] and malaria [6]. Successful POC testing allows for quick diagnosis and further management decisions (referral, follow-up testing or treatment) completed in the same clinical encounter or at least the same day, while the patient waits [7]. Yet, the mere availability of a rapid, cheap or simple test that can be conducted outside laboratories does not automatically ensure the POC continuum. Sometimes tests get misused or underused [8] or they require additional infrastructural, financial, and operational support [9]. Results of HIV rapid tests in South Africa, for instance, are sometimes not used to impact treatment decisions in a timely manner [10]. Challenges remain after a positive result with linkages to care [11] and follow-up testing [10]. It matters how tests are used in POC testing programs, which might employ different technologies, users and purposes across homes, communities, peripheral laboratories, clinics, and hospitals [7]. This means that testing needs to fit into a variety of existing workflow and care processes. An understanding of the diagnostic practices across settings is needed to develop, evaluate and scale up POC testing programs successfully.

However, the majority of the literature on POC tests does not examine how tests are used. Much of current research focuses on cost-effectiveness, feasibility, and acceptability of POC testing in one specific healthcare setting [4, 12–16] or uses clinicians’ concerns regarding POC testing as potential indicators for uptake [15, 17]. Research on diagnostic processes, the role of POC tests for clinical and patient decision-making, and the adaptation of POC programs to various socio-cultural contexts is limited. Such insights would allow understanding health system requirements and impact of technologies on diagnostic processes and delays [18, 19].

For stigmatized infections such as HIV, the difficulties of patients’ accessing diagnostics have been well documented (f.i. [20–22]), as well as the challenges in protecting human rights, informed consent and ensuring linkages to care [23, 24]. For HIV rapid testing in particular, the literature explores reasons for seeking testing and a (delayed) diagnosis (f.i. [25–27]), as well as factors enabling and deterring uptake of HIV testing [28]. Yet, few studies explore how the social and health systems contexts in which providers, health facilities and their diagnostic processes operate influence HIV testing practices [29–33]. These studies show that counseling is crucial for care-seeking and treatment adherence [31, 32]. Counseling practices differ across providers [30] and are prone to power imbalances between patients and providers [29].

While counseling is a crucial part of diagnosing HIV at POC, diagnostic practices also encompass ordering and conducting the test, reading the result, acting on the result and initiating follow-up steps. Anthropological work on malaria rapid tests highlights the importance of social relations for diagnostic practices [8]. The over-diagnosis of malaria in Tanzania is caused by multiple social influences, such as perceived expectations from colleagues, training that promotes the importance of malaria, pressure to conform with perceived patient needs and quality of diagnostic support [34]. Aside from social relations, material and infrastructural aspects are central for diagnostic practices. Lacking effective management of human resources and uninterrupted supply of clinical materials, impede the timely diagnosis of tuberculosis in South Africa [35].

This paper aims to understand the practices involved in diagnosing HIV at POC in South Africa and the implications for ensuring the POC continuum. The analysis is based on a larger qualitative study that examines diagnostic practices and challenges across major diseases and actors (doctors, nurses, community health workers, patients, laboratory technicians, policymakers, hospital managers and diagnostic manufacturers) at different points of care in South Africa (homes, communities, clinics, peripheral laboratories, hospitals). This approach allows paying attention to social relations, material and infrastructural aspects along the different steps of diagnosing. In a first analysis, we examined where POC testing of major diseases is successful and what the main challenges are. Amongst others, we showed how providers’ responses to delays due to laboratory-based testing result in yet additional delays and put strain on workload, human resources and patient interaction [36]. Here we zoom in on the widely used peripheral testing for HIV with rapid tests and focus more specifically on the strategies of providers and clients to overcome challenges in HIV testing at POC. While the majority of testing in South Africa is centralized, decentralized HIV testing offers important lessons for POC testing programs. The strategies to deal with challenges are not usually documented by public health data or analyzed in the biomedical literature on POC diagnostics. Yet, they are crucial for our understanding of diagnosing at POC and therefore the contexts within which diagnostic devices and policies need to function.

### Theoretical considerations

A focus on diagnostic practices to understand challenges of POC testing, as well as strategies to overcome these, is necessary if we view implementing diagnostic tests as an ongoing process. The settings and the test have their own histories, assumptions, practices and understandings inscribed in them. This means that by implementing tests successfully both the setting, including its organization of the workflow, workforce, its infrastructure, interaction with patients and standards, and the tests are being shaped and need to be adapted. In this co-construction of tools and (user) practices [37], the material dimensions of diagnosis, such as the test platform, reagents and supplies, the actors involved, their relations and the socio-cultural context in which testing and diagnosis is happening are invariably interlinked [38, 39]. Those aspects thus need to be studied together.

Here we focus on what providers and patients do to make HIV POC tests work and how they manage constraints. Inspired by the concept of tinkering strategies by Knorr-Cetina [40], we highlight the different adaptive strategies that are necessary to overcome specific challenges. Casper and Clarke discussed multiple tinkering strategies that over time turned pap smear, a rather recalcitrant diagnostic test, into a major cancer screening technology and the right tool to do the job. Some of these strategies for managing constraints are portable, meaning they can work anywhere (f.i. gendering the division of labour, automating the laboratory), while others are embedded in local practices (the local smear classification systems are based on routine interaction between clinicians and labs reading a particular sample and a woman’s history) [41].

Making diagnostics work also implies making the intervention fit with different understandings of local reality. During implementation of a national CHW policy in South Africa, all policy actors exercised a different form of power to make the intervention fit their understanding. This had consequences for the implementation of the policy [42]. We will therefore pay attention to the different rationales underlying providers’ and clients’ adaptive strategies.

## Methods

This paper is based on data collected during a qualitative research project with the aim to examine barriers to POC testing between September 2012 and June 2013 in two South African urban settings (Durban and Cape Town) and a rural one (Eastern Cape). Cape Town and Durban have a combined population size of approximately 5 million. The Eastern Cape is less densely populated, with 6.5 million spread out over 168,966km^2^. Clinics in the Eastern Cape are fewer than in the urban study settings and often far away with only very few ambulance services. Facilities are often poorly equipped, lacking human resources, medication and supplies [43]. Accessing healthcare is further compounded by poor water and sanitation, insurance status and low socio-economic status of people [44].

### Study design, data collection and participants

The data collection included 101 semi-structured interviews with doctors, nurses, CHWs, patients, laboratory technicians, policymakers, hospital managers and diagnostic manufacturers. Participants were purposively sampled to represent the settings of hospitals, peripheral labs, clinics, communities and homes in both the public/private sector and rural/urban setting (Table 1). Additionally we conducted seven focus group discussions (FGDs) with TB patients, nurses and CHWs, all of which were selected on a convenience basis to represent different perspectives. TB patients were specifically selected due to the overall project’s aim to draw implications for the development of new TB tests. The total number of FGD participants was 40, with a median group size of 5.7. In the context of conducting interviews and FGDs, we visited labs, clinics and testing facilities.

**Table 1:** Participant overview per setting

Setting	Type of participant	No. of interviewed participants (interview code)				Total interviews	No. of FGDs (FGD code)
		Urban Public	Urban Private	Rural Public	Rural Private		
HOME	TB patients (TB) and diabetic patients (DP), HIV patient (HIV), general OPD patients (OPD)	DP#1, OPD#1, 2, TB#1	0	DP#2,3, HIV#1	0	7	FGD#1_TB, FGD#7_ TB
COMMUNITY	Community Health Worker (CHW); NGO TB coordinator (NGO), NGO nurse (NN)	CHW#1, 2, 3, 6, 7, 8, 9, 10, 11, NGO#1, 4,5 NN#1, 2,	0	CHW#4, 5, NGO#2, 3	0	18	FGD#2_CHW, FGD#3_CHW, FGD#4_CHW, FGD#6_CHW
CLINIC	Private practitioner (PP); clinic nurse (CN), clinic doctor (CD), Public clinic manager (PCM), Faith healthers (FH)	CD#1, 2, CN#1, 2, 3, 7, 8, 9, PCM#1, 2, 3,4, 5, FH#1	PP#1, 2, 3, 4, 7, 8, 9	CN#4, 5, 6, FH#1	PP#5, 6	27	FGD#5_nurses
PERIPHERAL LAB (stand-alone)	Lab technician (LT); Lab manager (LM); NHLS consultants (NHLS), microbiologist (M)	NHLS#1, 2, LT#1,2, LM#1,2, 9, M#3	M#2, LM#8, 10	M#1, LM#6, 7	0	14	
PERIPHERAL LAB (attached to clinic)	Lab technician (LT); Lab Manager (LM);	LM#3	0	LM#4, 5	0	3	
HOSPITAL	Specialist molecular biology (SMB), −chest (SC), −dermatologist (SD),- internal medicine (SI), − infectious diseases (SID), −nutrition, (SN), −research coordinator (SRC), − health economist (SHE),	SMB#1,2, SC#1,2, SRC#1, SD#1, SHE#1, SIM#1, SID#1	0	SN#1	0	10	
	Hospital Manager (HM);	HM#1, 4, 5	0	HM#2, 3	0	5	
	Professional Nurse (PN), Research nurse (RN), Hospital doctor (HD),	RN#1, 2, PN#5, 6, 7, 8, HD#4, 5	0	PN#1,2,3, 4 HD#1,2, 3	0	15	
DIAGNOSITIC COMPANY	Managers from industry (MI)	MI#1,2	0	0	0	2	
TOTAL						101	7

The topics explored during the interviews included diagnostic processes for the major diseases per setting (mainly HIV, TB, diabetes mellitus, diarrhoeal diseases and hypertension) and challenges therein, understanding of diagnosis, and visions of an ideal test. For this paper, only the data on HIV was used. The interviews specifically examined diagnostic steps for each major disease occurring in the setting in great detail from ordering a test to acting on a result, including available material and capacities, turn-around-times, and referral processes. The FGDs focused exclusively on challenges experienced when diagnosing. Interview and FGD guides (see Additional files 1 and 2) were piloted and revised during the fieldwork to improve clarity of questions. All interviews and discussions were held in English and digitally recorded, and the note taker wrote down main points raised, non-verbal communication and setting characteristics.

### Data analysis

Audio files and notes were transcribed and cross-checked. Data analysis was done using Nvivo 9 (QSR International). We devised a coding scheme, which was tested on a handful of varied interviews and further refined [45], and coded the material in close communication with each other, further grouping material into emerging topics and themes in an iterative manner [46, 47]. The analysis is based on writing memos and thick descriptions of diagnostic practices and challenges per setting and disease. In addition to the deductively created themes according to the research questions (f.i. steps involved in reaching a diagnosis), additional subthemes were identified inductively (f.i. interactions with other providers and patients, strategies to overcome challenges). For this paper, the analysis is based on data on HIV testing processes in clinics, hospitals and communities. Professional roles are used to mask study participants’ identity.

## Results

At the time of this research, the South African HIV guidelines emphasized both provider-initiated testing and counseling and voluntary counseling and testing [48]. Nurses, community health workers (CHWs), counselors and some private doctors routinely use HIV rapid tests in clinics, within communities and in hospital wards. A drop of blood is taken through finger prick and added onto a testing strip (lateral flow assay) along with some reagent. The results can be read off the testing strip after a specified amount of time (f.i. 15 min). If the rapid test result is positive a confirmatory rapid test is conducted by the same provider. If the results of these two initial rapid tests are discordant, a nurse draws blood for a laboratory-based ELISA test. The client is asked to come back to the clinic within 1–2 weeks for ELISA results. If both initial rapid tests are found positive, the client is referred to a nurse or doctor who screen for TB and order a lab-based CD4 count that indicates functioning of the immune system and progression of the virus. If the CD4 count is below 350, indicating advancing HIV, treatment for HIV will be initiated by a clinic doctor.

This means that despite availability of HIV rapid tests in clinics, actual treatment initiation for HIV happens only 4–7 days after initial diagnosis and 3–4 days after a low CD4 count was diagnosed, because of laboratory-based follow-up tests and counseling sessions (CN9). This can take longer in hospitals where follow-up testing for urea, electrolytes and liver function takes another 1–2 days in addition to five adherence counselling sessions. Finally, treatment is initiated during the monthly or biweekly treatment days (HD2). Thus, even though clients are diagnosed as HIV-infected within one clinical encounter, treatment initiation in hospitals often only starts one month after initial diagnosis. The risks associated with these delays are that patients might not return, are lost to follow up or develop advanced stages of disease. Furthermore, constraints in equipment, spaces, lacking human resources and excess workload can challenge POC testing (FGD5_nurses; LM3; NHLS2; HD4; HM3, 4, 5; HD2, 4). In what follows, we discuss the strategies of providers and clients to overcome these constraints and to make HIV testing successful or worthwhile.

### Overcoming constraints in equipment, spaces, human resources and workload

In order to successfully diagnose HIV at POC, providers need to adapt to available infrastructure, material and spaces. This has consequences for the quality of results and requires providers to be flexible in their diagnostic practices, build relationships with others or adapt self-sustaining strategies.

#### Maintain relationships

Hospital managers, laboratory technicians, doctors and nurses emphasize the importance of maintaining relationships with other providers to overcome stock outs of testing material and manage the high workload. HIV test kits can regularly be out of stock, particularly at rural clinics (HM2; NGO2; FGD4_CHW). Through maintaining close personal relationships to clinic nurses, rural hospital doctors or NGO, staff who regularly visit clinics can help out with equipment (HD2; HM2). A program manager explains why she drives out to a remote clinic to deliver HIV test kits:

*So even on Monday, I drove out to […] clinic, which is about you know a forty-five minute drive each way and I went purely to take their test kits, because I found out on Monday, that they have been without test kits for a week or two. (NGO2)*

Similarly, urban NGOs borrow HIV testing material from each other when they are not supplied (NGO4), while others emphasize the importance of maintaining a good relationship with the Department of Health to receive stock on time through communicating and completing statistics and reports (NGO5).

Relationships in combination with self-sustaining strategies are also necessary to overcome absent or dysfunctional communication infrastructure to access laboratory results. Providers highlight that in rural clinics, accessing laboratory results rapidly is often impeded by absent phone, fax, or internet (PHN2; HM3; HD1, 2, 3; LM4). We met several nurses who use their own mobile phone and airtime to call the laboratory or hospital (LM4). Rural hospitals or clinic doctors then call back the nurses to save their airtime or go to the laboratory in search for the test results.

In many clinics, rural and urban, CHWs and nurses told us they rely on referring clients internally to each other to cope with the high workload (FGD4_CHW; HD2). Depending on the clinic, CHWs’ main function is to test for HIV (in which case they are called counselors), trace TB patients in the communities or assist the nurses. Counselors are also asked to translate for doctors, assist the nurse in testing, or find patient records, often while they are in the middle of a counseling session or during their break. They highlight that these diversions are difficult to refuse but disrupt their relationship with clients (FGD2_CHW). CHWs complain that in some clinics nurses refer so many clients that CHWs test 20–30 clients for HIV per day instead of 10–12. This has consequences for the quality of testing. CHWs admit that they are tired and cut short counseling time, while clients have waited too long and are impatient or aggressive (FGD2, 6_CHW). The social hierarchies present between CHWs and other healthcare providers imply that sometimes tasks are shared unequally. This task-shifting rather than task-sharing can put a strain on relationships among providers and between clients and providers.

The high staff turnover common throughout the public sector does not allow building trust-based relationships which would ease such modifications and encourage staff ownership of work (SRC1; RN2). What is more, HIV testing targets can lead to competition between nurses and CHWs over clients further eroding these relationships (CHW2, 3). The relationships between providers matter for POC testing and are shaped by it.

#### Adapt testing guidelines and practices to stock-outs

Due to stock-outs and limitations in supply, HIV test kits change frequently. A nurse in a rural clinic adapts the testing guidelines when she is out of HIV rapid tests. She then uses the confirmatory test first and if positive sends samples for ELISA for further confirmation. This adaptation prolongs the turn-around-time by a week and can confuse clients because different tests are used (CN5).

While the HIV rapid test kits have standardized protocols and operating procedures, they all differ slightly in, for instance, steps to perform, amount of reagent used or time to wait for results. Our study participants agree, that the frequent changes of test kits and procedures create room for error, especially in the absence of stringent quality assurance measurements (NGO2; FGD3, 4_CHW). Furthermore, most staff is trained for one particular test kit only (NGO2, 5; PCM1; CHW9). A hospital manager highlights:

As a consequence, all counselors we talked to described different ways of using the test kits. Some described waiting 15 min before reading results as a way of assuring quality of testing (CHW8, 9). Others would read results earlier:

*..I know a lot of people are quite impatient and with lots of patients waiting, they do not always wait the full 15 min, so I have had a few negative tests, where I am like, this person is definitely positive and you do it again and it only comes up positive later like in 10 min or 12 min. (HD1)*

Either to accommodate impatient clients or because, based on their experience, results tend to show up earlier given a particular temperature and season (HM3; HD1).

*If someone is in a hurry, we can actually do everything within 5 min.*

*INT: And how long does the test run?*

*The test runs, its actually strange, in winter, when it’s cold, it actually really runs slower. In summer, I do not know how, but in summer it actually runs very quick, but we do not give your result before 5 min. (NN2)*

These adaptations in practices have unclear implications for the quality of testing. They are in stark contrast with the detailed instructions for performing tests foreseen by test developers. For instance, wrong handling of the finger prick can influence the outcome of the test. A program manager explains some of the details they teach the counselors:

A lot can go wrong with this seemingly simple procedure, for instance, choosing the wrong finger, not holding the finger correctly, shaking, not producing sufficient sample size, milking the finger and squeezing tissue fluid out or producing air bubbles that interfere with the blood sample. Test developers complain that operator errors happen because of improper training (MI1, 2). Instead, a CHW describes the difficulty of drawing blood, including personal preferences for certain test kits:

*Like for example there is a certain way you need to hold the persons hand and sometimes when the person is shaky, you [be] come shaky [laughing]. And the capillary tubes, I did not like the plastic ones; I preferred the glass type capillary tube (…) because we drew up the blood easier. (CHW2, 3)*

To the various users at POC, precision means adapting testing to the context of season, material availability and personal preferences. Precision thus means different things to test developers and users.

As a consequence of these adaptations to material constraints, providers mistrust the quality of care provided by other providers and in response compound further delays. A doctor in a rural hospital reveals that sometimes he would test clients’ knowledge to check prior counseling done by nurses or CHWs (HD2). In one hospital we visited, the HIV rapid test was removed from the ward back into the laboratory to avoid multiple staff completing tests. If the laboratory was busy the turn-around-time could extend from 15 to 20 min to hours or even days (SRC1).

#### Adapt testing guidelines and practices to physical space

Adapting to the physical space within which testing happens influences the quality of a diagnosis. Each time when testing in communities, CHWs have to get used to a new space, such as malls, tents on roadsides, prisons or rooms in businesses, universities and schools (CHW2,3,6, 7; NGO5; NN1). CHWs highlight how they improvise in these temporary spaces with limited means to ensure confidentiality and comfort to clients seeking a HIV diagnosis.

If the space is too cold, wet, drafty, neither comfortable nor confidential, the CHW and the client might not be relaxed and diagnostic quality suffers: The clients might not reveal all information and the CHWs might not question and probe as thoroughly as they normally would. Instead, the CHWs we talked to will try to keep their voices low, cut short the counseling and not take blood samples for further testing. These follow-up steps would take an additional 20 min and might reveal a positive test result to other waiting clients (FGD6_CHW). Instead CHWs refer these clients to follow-up counselling at the clinic (CHW4; NGO4).

*You can see when a client is not comfortable. I am gonna cut everything (…) short because in most cases when its public event you (…) end up referring that person to a clinic where you just gave the basic and that’s it. Hoping that person’s gonna end up at a clinic, you understand, go for a confidential follow-up session. (R1, FGD6_CHW)*

The space can also determine the time available for testing. According to CHWs, in outreach settings this time is often limited and clients are not always willing or able to wait 15 min for results. To save time, CHWs do the pretest counseling for an entire group (CHW6, 7; NN1).

### Actively managing diagnostic processes

In varied ways, clients take an active role in managing their diagnostic process including attempts to control where they receive testing, how and which results they are able to obtain. Providers try to support clients in accessing health services. These strategies in turn create new challenges for diagnostic practices in settings with limited (human) resources, functioning referral systems and high workloads. In making tests accessible the diagnostic test is repurposed by both clients and providers.

#### Adapt testing practices to different clients

In order to ease cost, long distances, limited clinic opening hours and transport infrastructure for clients, providers arrange for follow-up testing at clinics closer to clients’ homes (HD1) and rural clinicians transport clients themselves or provide transport money (HD1). Some providers mentioned providing services on weekends (PCM2) or arranging that a client is managed completely in one centre or in one visit day (CD2; RN1). To help clients overcome the barriers of stigma and discrimination in accessing services, some providers highlight that they offer to meet for testing at agreed locations to avoid clients’ home community (FGD2_CHW), will have a nurse conduct the test if the client knows the CHW (CN5), or accompany clients who have tested positively in the community to the clinics for accessing treatment (NGO4, 5).

During the HIV testing process, CHWs reported to continuously adapt their consultative practices to respond to crucial socio-economic problems clients might have. These often go far beyond the testing procedure and can include marital counseling or crisis management which are not accounted for (CHW2, 3; RN1; FGD2, 6_CHW).

*…a person comes to us and they talk about their marriage and all that other things. You cannot say to the person “look I’m not trained in that”. (…) should the person come with a drug problem, at least we can talk to the person a little bit or maybe refer the person to whoever or wherever. (*CHW3)

These adaptations in testing practices and processes are only possible if there are sufficient human resources and a reasonable workload. Furthermore, they rely on a functioning referral system within and between centres. Yet, these aspects are often lacking, risking loss of clients from testing pathways.

#### Turning the test into a tool to reach another aim

Providers reported that clients actively chose their point of testing in a way that fits their lives; this includes considerations of cost and distance to reach the clinic, but also of avoiding social stigma (NHLS1; NGO2, 4). Similarly, if the guidelines for accessing a certain treatment or testing procedure do not fit the client’s circumstances, some who have been tested positive before present as new clients; they either stop their treatment or hide their HIV status during the counseling session. Some clients do so to access the HIV single pill treatment, which is only available to newly diagnosed clients, pregnant and breast-feeding women (CHW4; PCM2). Others want POC CD4 monitoring which is only offered to new clients by certain NGOs (NGO4; CHW6, 7; NN1). In that case, clients use a positive test result to advance in the testing process and access another device, the POC CD4 count monitor. Providers also reported examples of clients presenting at clinics with fake identification cards, either to obtain a negative result to find employment or help out a friend or to obtain a positive result to access a government grant (CHW6, 7; NN1; NGO4). This puts an extra burden on the health care system because additional testing resources are used and creates difficulties with follow-up and tracing (NN2).

Similarly, providers realized that clients actively test the quality of HIV testing. Some clients access private clinics to confirm a rapid test result obtained elsewhere with a laboratory-based test (FGD6_CHW). Other clients test CHWs by returning for repeat testing, to see if tests still give the same result (FGD2, 6_CHW; NN2). Again others ask to retest after having seen a priest promising cure from HIV (FGD2_CHW). This retesting might produce negative results, according to the CHWs because the virus can be undetectable due to ongoing antiretroviral (ARV) treatment.^1^ This creates confusion and puts CHWs in a difficult position to explain divergent results and why a negative test result does not mean cure (FGD2_CHW; NN2; NGO3).

*Sometimes people who have already tested and know that they are positive will come and hope for another result. Sometimes patients who have been on ARV treatment for a long time will test negative. These people wish that the virus is gone. It can be daunting if the person does not disclose their status beforehand. (R1, FDG6_CHW)*

CHWs discussed multiple strategies of finding out whether a client tested before or is on ARV treatment. Yet, the extra probing required takes time and is not possible in all testing locations (FGD6_CHW).

In this way clients actively turn the test into a tool to achieve another goal: change the treatment, access a certain follow-up test, acquire another test result, or confirm an act of god/alternate medication. These repurposing acts can create new challenges to POC testing.

#### Turning the testing process into a tool to enhance adherence

Providers use the testing process to convince clients to accept their illness and adhere to their advice (FGD6_CHW). During the HIV testing process, clients essentially self-diagnose by reading their own results off the testing strip (CN6). This is done to avoid providers stating a wrong result, in case the client is still in the window period, but also to enhance a client’s ownership over the test result and thus ease acceptance.

*It is the client whose gonna read his or her results, (…) you showed the test to the client, It’s clean, there’s nothing until you put that blood sample and the buffer and you put it in front of the client, and …(…) it’s the client whose gonna say, ‘but I have seen everything. The whole process was done in front of me. (…) whatever it’s gonna be, these are the results. I must accept it because I was part and parcel of the whole process. (NGO3)*

Yet, that does not always solve the problem. According to CHWs some clients have made up their mind that they are negative, while others resort to purposively spreading the disease to avoid dying alone (FGD 3_CHW). Sometimes test results create confusion and reinforce clients in not-accepting the result. This happens in cases of divergent results between the first and confirmatory rapid tests, when a confirmation test by ELISA is necessary or when test kids have changed (NN2; FGD4_CHW).

*..for the clients who are repeating the test, because she knows that I was here and I used this kind of test kit and now I am using this, so it can be easy for the client to say, ‘no it’s your kit that is wrong’. (FGD4_CHW)*

In those cases the test and testing procedures are mistrusted and blamed for producing wrong results. In response, some providers discuss how they show the expiry dates, new lancets and gloves to clients (CHW6, 7; NN1). To ease acceptance, a clinic nurse repeats the tests up to four times and prolongs the counseling session or orders laboratory results to back-up rapid test findings (CN6).

## Discussion

When testing for HIV at the POC in South Africa, providers and clients employ different strategies to overcome constraints in equipment, spaces, human resources and workload and to make testing accessible, worthwhile and successful.

Providers overcome material stock-outs by maintaining relationships with other providers, by developing self-sustaining strategies or by adapting testing guidelines and practices. To overcome limitations of testing facilities and spaces, CHWs cut short counseling and testing practices and improvise with limited means to ensure confidentiality. Providers respond to limited transport infrastructure and stigma-related concerns by adapting opening hours, testing locations or staff and counseling practices. These adaptive strategies are locally negotiated solutions, often ad-hoc, depending on personal commitment, relationships, available human resources and functioning referral systems; all of which is often absent.

Some strategies can prolong diagnostic turn-around-times. We showed elsewhere how calling laboratories for lost results, a strategy to overcome logistic challenges and resource constraints, is adding to further diagnostic delay by disrupting laboratory work [36]. At times providers need to adapt testing guidelines, a common practice among healthcare providers. Research on occupational therapists in the Netherlands showed that while these professionals were not following the guidelines, they still acted in the spirit of the guidelines [49]. The nurse who adapts the HIV testing guidelines to overcome stock-outs acts in the spirit of the guidelines or what she perceives to be the core recommendations [50], namely clients should be tested on the spot. However, this can create confusion among clients or delay the POC continuum.

These adaptive strategies can also lead to frictions and mistrust between nurses, doctors and CHWs if guidelines are not followed, testing processes are adapted or workload is shared unequally. The latter particularly happens when relationships are hierarchical, with consequences for quality of testing, in which case relationships to clients also suffer. Social relationships between providers and between clients and providers can thus change due to testing. The importance of social relations for use of diagnostic tests at POC and decision-making on test results has been underscored before [8, 34]. Our study shows that relationships are also crucial for ensuring material supply and overcoming stock-outs, e.g. for the logistics of POC testing. The role of logistics of diagnosing in the health system of South Africa is often overlooked [35].

The physical space matters for HIV testing as it can in/decrease confidentiality and stigma (f.i. [51]). Beyond that, we show that the physical space has implications for the quality of the diagnosis. Depending on the space, CHWs cut short counseling, fail to encourage an open discussion or do not conduct all necessary tests. Research on telecare technologies showed [52] that the place in which technology, in this case the HIV rapid test, is used enables or constrains interactions between actors.

In making HIV testing work at POC, the strategies involved require flexibility in professional roles and diagnostic practices of nurses, doctors and CHWs. In doing so some need to go beyond what is inscribed in the guidelines or their job profile, while often the test and testing process is repurposed by the clients and providers involved. Clients use the diagnostic services to pro-actively manage and monitor their own condition. Similar to the observation that accessing treatment can function as an incentive of testing [31], clients use a particular test result to change the treatment, access a specific follow-up test, gain a grant or employment or test the quality of diagnostic services. In this way, clients pro-actively manage their condition and circumvent guidelines. Diagnosing HIV at POC can empower clients. Providers use the testing process as a tool to convince clients to adhere to their advice. These different adaptive strategies have their rationale in the provider and client’s efforts to make the diagnostic tool fit their understanding of local reality. Casper and Clarke emphasize how the ‘rightness of the diagnostic tool’ pap smear is constantly constructed by actors with different perspectives, interests and agendas on what the job is that needs to be done [41]. What matters here is not so much the rightness of the tool, but the multiple roles of the diagnostic device in completing diagnostic processes and their implications for the POC continuum in resource-constrained settings dealing with a stigmatized disease.

Overall, not all of these repurposing acts and adaptive strategies are successful in ensuring a POC continuum. Some lead to disruptions, unnecessary testing or delays. These results also show that testing in the community and involving clients and non-technical staff in the testing process can have unclear implications for quality of testing. Precision, for instance when pricking a finger to draw a blood sample, is practiced differently in the laboratory and everyday testing of CHWs. Test results and testing processes take on new meanings for clients and providers at POC. HIV testing technologies are thus fluid, they take on different shapes depending on the providers and clients involved [30]. At the same time, tests shape relationships, professional roles and practices of users at POC.

## Conclusion

Examining diagnostic practices involved in HIV POC testing offers important lessons for development of future POC testing programs. This analysis examines the strategies of healthcare providers and clients to overcome infrastructural, economic and socio-cultural constraints in HIV POC testing in South Africa. We showed that testing processes are dynamic in response to varying client needs, resource constraints and available materials. The focus on adaptive strategies allowed to move beyond examining challenges of testing HIV at POC and instead revealed the ad-hoc, fragile and unsustainable nature of these strategies in settings lacking human resources, testing equipment and functioning referral systems. These strategies have consequences for the relationships among providers and outcomes of HIV testing. While most adaptation is testimony to professional flexibility and personal commitment, some strategies delay POC testing, lead to frictions between providers and compromise quality of diagnosis.

## Additional files

Additional file 1: add information_interview guide: Interview guides for provider and patient interviews. (DOC 74 kb)

Additional file 2: add information_focus group guide: Moderator guides for focus group discussions with medical officers, nurses, doctors; community health workers and patients. (DOC 56 kb)

## Abbreviations

ARV: Antiretroviral
HIV: Human immuno-deficiency Virus
POC: Point of care
TAT: Turn-around times3

## Footnotes

1 This goes against established clinical knowledge: HIV rapid tests are testing antibodies and those should always be present despite ARV treatment. False negatives should be a very rare occurrence and only happen in cases where ARV therapy was started soon after exposure to the virus and the body did not have time to establish a full antibody response.
