# Supplementary material for: Making HIV testing work at the point of care in South Africa: a qualitative study of diagnostic practices
Source: BMC Health Serv Res. 2017 Jun 17;17:408. doi: 10.1186/s12913-017-2353-6 (PMC5473989; doi:10.1186/s12913-017-2353-6)
Supplement: Supplementary file 2 — add information_focus group guide: Moderator guides for focus group discussions with medical officers, nurses, doctors; community health workers and patients. (DOC 56 kb) [file 12913_2017_2353_MOESM2_ESM.doc]

**Focus group discussions POC testing South Africa**

The aim of the focus group is to obtain in-depth views with regard to the following topics:

- To understand potential needs or concerns of the different groups for POC testing
- To understand why the needs exist
- To collect ideas about possible solutions for POC testing in different settings

The aim is not to obtain consensus on these topics, but to map ideas and feelings and to explore the problems and the solutions.

**Moderator guide: Medical officers, nurses, doctors**

1. **Introduction:**

- By the moderator:
  - purpose of session
  - recording& confidentiality
  - ground rules of group discussion(It will be explicitly stated that there are no wrong and right answers and that the study is explorative and not intended as a clinical audit or assessment)
- Participants introduce themselves(if necessary use of name cards)name, profession/ affiliation

1. **Predisposition phase:** To establish what particular problems participants experience or define with regard to diagnosing infectious diseases at their point of care

- What are the biggest challenges that you face when diagnosing infectious diseases in your setting?
  - - Short silence in which participants write down ideas; one challenge per post-it
    - Moderator groups post-its on the wall/board (and clarifies any unclear notes/words)

1. **Group discussion on the following topics (probe for subpoints if they are not covered):**

- Challenges encountered with diagnosing infectious diseases in your setting?
  - Discuss grouped challenges one by one as listed by participants
  - Discuss their explanations for reasons of challenges
- What can be done to improve this situation?
  - What could be solutions?
  - Who would be responsible
  - How feasible are these solutions?
- What would a new diagnostic test need to work in this setting?
  - TAT, timing, work-flow
  - cost, incentives,
  - policy, regulation,
  - infrastructure, laboratory capacity, supply chain,
  - quality control,
  - training,
  - infection risk,
  - reporting, administrative/operational,
  - awareness, socio-cultural factors
- User feedback on dummy POCT (pass around dummy or pregnancy/rapid Malaria/HIV test or concept statement explaining our definition of POC (POC defined as rapid feedback system of results to allow a decision to be made in the same clinical encounter can be from minutes till end of the day))
  - overall reactions
  - strengths & limitations
  - comparison with current device
  - usability, feasibility Why? Why not?
  - Barriers to use

1. **Summary**

- Moderator summarizes results
- Short survey among participants whether they have comments or anything to add

1. **Debriefing (discuss any comments on the process of the FGD as such)**

**Moderator guide: Community Health Workers**

1. **Introduction:**

- By the moderator:
  - purpose of session
  - recording & confidentiality
  - ground rules of group discussion(It will be explicitly stated that there are no wrong and right answers and that the study is explorative and not intended as a clinical audit or assessment, that there is a quiet phase where we ask a question, speaking one by one rather than all together)
- Participants introduce themselves, name, scope of activity

1. **Predisposition phase:** To establish what particular problems participants experience or define with regard to diagnosing infectious diseases at their point of care

- What are the biggest challenges that you face when diagnosing infectious diseases in your setting?
  - - Short silence in which participants write down ideas; one challenge per post-it
    - Moderator groups post-its on the wall/board (and clarifies any unclear notes/words)

1. **Group discussion on the following topics (probe for subpoints if they are not covered):**

- Challenges encountered with diagnosing infectious diseases in your setting?
  - Discuss grouped challenges one by one as listed by participants
  - Discuss their explanations for reasons of challenges
- What can be done to improve this situation?
  - What could be solutions?
  - Who would be responsible
  - How feasible are these solutions?
- What would a new diagnostic test need to work in this setting?
- What would a new diagnostic test need to work in this setting? Probe for:
  - TAT, timing, work-flow
  - cost, incentives,
  - policy, regulation,
  - infrastructure, laboratory capacity, supply chain,
  - quality control,
  - training,
  - infection risk,
  - reporting, administrative/operational,
  - awareness, socio-cultural factors
- User feedback on dummy POCT (pass around dummy (POC defined as rapid feedback system of results to allow a decision to be made in the same clinical encounter can be from minutes till end of the day))
  - overall reactions
  - strengths & limitations
  - comparison with current device
  - usability, feasibility Why? Why not?
  - Barriers to use

1. **Summary**

- Moderator summarizes results
- Short survey among participants whether they have comments or anything to add

1. **Debriefing**

- discuss any comments on the process of the FGD as such

**Moderator guide: Patients**

1. **Introduction:**

- By the moderator:
  - purpose of session
  - recording& confidentiality
  - ground rules of group discussion(It will be explicitly stated that there are no wrong and right answers and that the study is explorative and not intended as a clinical audit or assessment)
- Participants introduce themselves(name, age, how long have you been diabetic, disease status & how you monitor it (diabetes), where you take treatment (TB))

1. **Predisposition phase:** To establish what particular problems participants experience or define with regard to diagnosing and monitoring their disease at their point of care

- What are the biggest challenges that you faced when **finding out about** your disease/ in the process until getting started on treatment/ and with **monitoring** your disease (diabetes)
  - - Short silence in which participants draw their ideas; one challenge per sticky-note
    - Moderator groups sticky-notes on the wall/board (and clarifies any unclear drawings with participants)

1. **Group discussion on the following topics (probe for subpoints if they are not covered):**

- Challenges encountered with **diagnosing** your disease?
  - Discuss grouped challenges one by one as listed by participants
  - Discuss their explanations for reasons of challenges
  - What can be done to improve this situation?
    - What could be solutions?
    - Who would be responsible?
    - How feasible are these solutions?
- Challenges encountered with **monitoring** your disease?
  - Discuss grouped challenges one by one as listed by participants
  - Discuss their explanations for reasons of challenges
  - What can be done to improve this situation?
    - What could be solutions?
    - Who would be responsible?
    - How feasible are these solutions?
- What would a new diagnostic test need to work for you? Probe for:
  - TAT, timing, work-flow
  - cost, incentives,
  - policy, regulation,
  - infrastructure, laboratory capacity, supply chain,
  - quality control,
  - training,
  - infection risk,
  - reporting, administrative/operational,
  - awareness, socio-cultural factors
- User feedback on dummy POCT (pass around dummy or pregnancy/rapid Malaria/HIV test)
  - overall reactions
  - strengths & limitations
  - comparison with current device
  - usability, feasibility by health worker/CHW? Why? Why not?
  - usability, feasibility by as self-testing device? Why? Why not?
  - Barriers to use

1. **Summary**

- Moderator summarizes results
- Short survey among participants whether they have comments or anything to add

1. **Debriefing (discuss any comments on the process of the FGD as such)**
